# First Principles of Surgery; Being an Outline of Inflammation and Its Effects

**Published:** 1837-07

**Authors:** 


### Art. XV.

*First Principles of Surgery; being an Outline of Inflammation and its Effects.* By George T. Morgan, a.m., Lecturer on Surgery in Aberdeen.—*London and Edinburgh*, 1837. 8vo. pp. 210.

All who feel interested in our profession must view with satisfaction the progress which the operative department of surgery has made during the last half century; but no educated surgeon will fail to recognize a far more worthy subject of congratulation and pride in the improved state of our knowledge in the treatment of disease, which renders operations less essential, and consequently less frequent. The labours of our illustrious countrymen, Cheselden, Pott, Hunter, Cline, and their contemporaries and immediate successors, have lent their powerful aid in dispelling the ignorance and prejudice by which the advancement of scientific surgery was so long retarded; and they have left the impressive lesson which, in this enlightened age, *we* ought not to forget,—that accomplished manual dexterity, though doubtless an indispensable qualification in the operating surgeon, should ever be held subservient to the far more desirable attainments which will enable him to have less frequent recourse to his scalpel.

Of all agents, whether in the production of disease or its removal, whether as the opponent or ally of the surgeon, no one has a more universally extended influence, or is more subtle and deceptive in its *modus operandi*, than inflammation. We need not wonder, then, that a more correct insight into the laws which regulate vascular action and the condition of the vessels under inflammation, should have had such a beneficial influence in establishing on a firm basis the principles of surgical practice. It is to the monographs of Hunter, Thomson, Gendrin, &c., that we are indebted for the most valuable contributions in this department of pathology. Let us hope that their example may induce others to labour in the same field of science, which still offers so ample a harvest to those who cultivate it carefully.

Mr. Morgan's work does not rank amongst the list of original contributions, but is essentially a student's book; his aim being "to render a simple statement of known or acknowledged facts;" and thus "to furnish the surgical student with an outline of inflammation and its effects, brought down to the present period." How successfully he has accomplished these objects will be best proved by a perusal of his work: in the mean time, we shall undertake the agreeable task of presenting our readers with a brief analysis of its contents.

A part of the Introduction having been devoted to a sketch of the course to be pursued in the subsequent investigation, the author proceeds to devote a section to the consideration of the arterial system, in which the opinions and experiments of Haller, Bichat, Hunter, Parry, Thomson, and other physiologists, are severally quoted and discussed. This is done with perspicuity and as much conciseness as the extent of the subject, and the many conflicting opinions to be noticed, will admit of; and the facts adduced, with their accompanying inferences, form an useful and interesting compendium of information which is absolutely essential to the right understanding of the subsequent sections.

In entering on the discussion of the *Theory of Inflammation*, the author seems fully impressed with the difficulties of the subject; and his introductory remarks are so just and apposite, that we are tempted to lay them before our readers: our quotation may further serve as a specimen of the simple and clear style, and, we may add, the philosophical tone of Mr. Morgan's work.

"To establish a just theory of inflammation is a point of the highest importance in medical science, as tending directly to improve our knowledge of morbid action, and enabling us to lay down precise rules of treatment, free from all appearance of empiricism. This is a subject still involved in dispute, and one on which even experimental research has failed to produce conviction. We enter upon it, therefore, more with the view of recording the phenomena we have observed, and making up our history of the disease, than with any hope of assisting in the adjustment of so intricate a problem. The difficulties we have to contend with, in watching the circulation in a state of health, apply themselves with double force when investigating the nature of inflammatory action; and, were our acquaintance with the former more enlarged, our knowledge of the latter would be comparatively easy. The more immediate phenomena of inflammation are confined to the minute blood-vessels, and to the changes going forward in them our attention must therefore be first directed. There is one way only of arriving at the truth, and that is by inducing the disease in the transparent parts of an animal, observing with the microscope the changes which ensue, and marking carefully the order of their occurrence. Nothing satisfactory can be elicited by considering the symptoms during life, or examining the alteration of structure left after death. On neither of these two heads can we reason correctly. From the first, any conclusions as to the state of the circulation must be vague and uncertain. From the second, still less can be gathered respecting the process of inflammation, because its effects alone are visible; the morbid action which led to them has ceased with life." (P. 33.)

Mr. Morgan enters upon his subject by a consideration of the two theories of increased and diminished action of the vessels; and his observations on the labours of Philip, Thomson, Hastings, and Kaltenbrunner, are judicious. After attempting "to amalgamate and reduce to a proper order the views entertained by the contending parties," the author dismisses his summing-up of the different hypotheses with a remark in which we cannot but concur, "that it is much to be regretted that some portion of the valuable time wasted on this subject has not been given to matters of greater importance."

In Section iii., the *local symptoms of Inflammation* are treated of, and the theory of the generation of heat is noticed. The same perspicuity of style is here evident, but nothing novel is introduced which requires remark. The consideration of the *constitutional effects of Inflammation* occupies the fourth section, and the subject is ushered in by a brief outline of the history of fever, comprising the theories which gave rise to the doctrines of the humoral and nervous pathology. The now-revived opinions of Hippocrates and Galen are first noticed, and the tenets of Boerhaave, Stahl, Cullen, Brown, and others, more fully discussed. Our author, however, does not rank himself with either the solidists or fluidists; for, whilst he adopts (justly we think,) the views of Andral with respect to *inflammatory fever*, which, as that pathologist remarks, "seems often to arise from no other source than the blood being too rich in fibrin," we find him subsequently expressing a hope that "the humoral pathology may emerge from the obscurity and ridicule thrown over it by its bigoted opponents, but, at the same time, that its due share in the constitution of disease may only be assigned to it."

The views of the later French pathologists regarding the origin of fever in *gastro-enteritis*, are next touched upon; and here Mr. Morgan is more explicit in the announcement of his own opinion. "Whether," he observes, "the primary source of common fever consists in a morbific poison applied to the blood in the lungs, and by which the nervous and other systems are subsequently affected, or whether the changes in the latter take precedence of the changes in the circulating mass, we do not pretend to determine; but we hold that the records of established cases coincide with daily experience in proving the worst varieties to occur without the coexistence or supervention of local disease." (P. 106.) Now, whether such local disease stand in the relation of cause or effect to the febrile action, we do not feel ourselves more satisfied than Mr. Morgan, although we acknowledge our leaning to the opinion, that the organic lesions which are found to accompany fever are often, if not always, the result of primary and local *functional* derangement; still we fully coincide with Broussais, Louis, and others, that the cases of fatal fever are indeed rare, in which organic lesion of some viscus may not be discovered, or where, if not discovered, it may not be inferred that our imperfect means of investigation is the true cause of such change having eluded our observation.

The symptoms of ordinary fever, and the morbid changes in the circulating mass, are next ably treated of, as is also the important subject of symptomatic or irritative fever. This section concludes with a vivid sketch of the two worst kinds of constitution in which a serious accident can occur.

"There are two principal morbid varieties of constitution in which local injuries produce peculiar and extraordinary effects. The one is that of general plethora attributable to over-repletion of the vascular system; the other arises from an impoverished state of the blood, coupled, in the worst species of cases, with a disturbed condition of the nervous system. We shall only select the extremes of these for examples, as it would be impossible to detail, within a brief space, the various combinations to be met with. Fortunately, such cases are easily recognized at the bedside, and their treatment must be regulated according to existing symptoms." (P. 144.)

The truth of the last sentence of the above paragraph we are ready to admit, but the propriety of the mode of treatment prescribed we must question. "The full, bloated habit," says Mr. Morgan, "is principally confined to those who live high and take little exercise, or those who eat freely and use large quantities of malt liquor," (p. 145): and again in the following page; " there is at first great excitement of the whole vascular system, followed by a corresponding degree of depression; there is a disposition to action, without sufficient strength to maintain it." Now surely this admission is scarcely consistent with the treatment that is there advocated. " Nothing at the commencement will suffice but free, general, and local depletion, with purgatives; and we have, by these means, known consciousness restored, after an unfavorable prognosis had been passed." (P. 147.) This is certainly at variance with the result of the experience of our best surgeons in the practice of the London hospitals. The cases are rare in which they can venture upon more than an active purge or topical depletion in a severe injury, such as compound fracture, in subjects of the class now referred to; and we have known the most disastrous consequences follow the use of the lancet, even in the most full, bloated habits. The student, therefore, should be warned that, although he may readily recognize such a condition of the system by the bedside, he must take into consideration the shock the frame has already sustained, and the demand there will be on the powers of the constitution to repair the mischief: and his experience will probably soon teach him that he will speedily be called on to afford support, in lieu of detracting from the animal powers. It is, however, proper to add, that the subjects of severe injuries, who breathe a pure air and follow a healthy occupation, as in the country and the smaller provincial towns, will bear and even require depletion; when the inhabitant of our densely populated cities and manufacturing districts would, under similar circumstances, sink beneath the remedy. The judicious practitioner will soon learn to modify his treatment with the varying circumstances of his patients.

The fifth section is on the *varieties in the process of inflammation;* the sixth on the *terminations* of inflammation; and in the seventh and last, the *progress of inflammation in different structures* is considered. This interesting and important subject is handled in a style quite equal, if not superior, to that in which the preceding divisions of the work are executed. Here, as elsewhere, Mr. Morgan availed himself largely of the labours of others, and has thus exhibited a clear and highly practical view of the character, progress, and results of inflammation in the various component textures of the body.

In thus presenting our readers with an outline of the contents of the volume before us, it will be perceived, that we have found but little to criticise and much to commend. If Mr. Morgan is occasionally rather severe in his strictures on the labours of others, his severity has its origin not in captiousness but in the more practical tendency of his own views, a tendency which cannot fail greatly to enhance the value of his book. We have already remarked upon the perspicuity of the style: there is also frequently a terseness and vigour of description which is very impressive, and effectually shuts the door against ennui. Altogether, the perusal of Mr. Morgan's little work has afforded us much satisfaction, and we can conscientiously recommend it to the surgical student, as an interesting and trustworthy guide to the most important of all the subjects to which his attention can be directed both in the commencement and in the progress of his labours.

